# Geometry Shapes Propagation: Assessing the Presence and Absence of Cortical Symmetries through a Computational Model of Cortical Spreading Depression

**DOI:** 10.3389/fncom.2016.00006

**Published:** 2016-02-02

**Authors:** Julia M. Kroos, Ibai Diez, Jesus M. Cortes, Sebastiano Stramaglia, Luca Gerardo-Giorda

**Affiliations:** ^1^BCAM – Basque Center for Applied Mathematics Bilbao, Spain; ^2^Computational Neuroimaging Group, Quantitative Biomedicine Unit, Biocruces Health Research Institute, Cruces University Hospital Barakaldo, Spain; ^3^Ikerbasque, The Basque Foundation for Science Bilbao, Spain; ^4^Department of Cell Biology and Histology, University of the Basque Country Leioa, Spain; ^5^Dipartimento di Fisica, Center of Innovative Technologies for Signal Detection and Processing, Istituto Nazionale di Fisica Nucleare Sezione di Bari, Università di Bari Bari, Italy

**Keywords:** cortical spreading depression, computational model, realistic cortical geometry, magnetic resonance imaging, finite elements simulation, reaction diffusion

## Abstract

Cortical spreading depression (CSD), a depolarization wave which originates in the visual cortex and travels toward the frontal lobe, has been suggested to be one neural correlate of aura migraine. To the date, little is known about the mechanisms which can trigger or stop aura migraine. Here, to shed some light on this problem and, under the hypothesis that CSD might mediate aura migraine, we aim to study different aspects favoring or disfavoring the propagation of CSD. In particular, by using a computational neuronal model distributed throughout a realistic cortical mesh, we study the role that the geometry has in shaping CSD. Our results are two-fold: first, we found significant differences in the propagation traveling patterns of CSD, both intra and inter-hemispherically, revealing important asymmetries in the propagation profile. Second, we developed methods able to identify brain regions featuring a peculiar behavior during CSD propagation. Our study reveals dynamical aspects of CSD, which, if applied to subject-specific cortical geometry, might shed some light on how to differentiate between healthy subjects and those suffering migraine.

## 1. Introduction

Migraine is a prevailing disease within a 15% of the world's population suffering from severe unilateral headache and nausea (Vos et al., 2012). About one third of migraine patients experience a migraine aura preceding the typical headache (Hadjikhani et al., 2001; Richter and Lehmenkühler, 2008). During this aura, patients undergo transitory perceptual, visual and/or auditory, disturbances. To the date, several studies and experiments suggest that a propagating depolarization wave on the cortex underlays migraine (see de Tommaso et al., 2014 and references therein). This wave of intense excitation, named cortical spreading depression (CSD), causes a drastic failure of the brain homeostasis and is followed by a wave of inhibition (de Tommaso et al., 2014). Starting in the visual cortex, CSD propagates to the peripheral areas in a time-scale up to 20 min (Leão, 1944, 1947). It is clear that wave propagations do drastically depend on the propagation medium (Sanides, 1962). In addition, the cortex geometry is highly individual and, to our knowledge, studies of CSD based on realistic (subject-specific) cortical geometries together with realistic neural modeling have not been addressed so far.

Several mathematical models of CSD have been used in the past, from reaction diffusion models to microscopic models accounting for the cells' connectivity. Based on the fact that the extracellular potassium concentration follows approximately the time-course of depolarized neurons and glia cells during spreading depression (Kraio and Nicholson, 1978), Tuckwell and Miura propose a model for its wave propagation in 1D heterogenous space. Including potassium and calcium fluxes, extracellular diffusion and active transport pumps in a Hodgkin-Huxley like system of equations, its numerical simulations portray the basic qualitative properties of the spreading depression waves and account for the annihilation of two colliding waves (Tuckwell and Miura, 1978). Reggia and Montgomery couple synaptic connectivity and extracellular potassium uptake at a single cell level in a simplified 2D array representation of the cortex (Reggia and Montgomery, 1996). They use a reaction diffusion equation to describe the potassium changes and project the simulated cortical activity onto the visual field to mimic the corresponding visual pattern. The potassium wave triggers irregular patches of highly activated areas on the visual field, supporting the theory of CSD underlying migraine aura.

A few recent studies started to consider the effects of cortical geometry on CSD propagation, at different levels of detail. Fissures and sulci of the cortex influence the propagation of depolarization waves and can stop the migraine in different positions depending on the patient. Pocci and collaborators studied the effect of the cortical bending by using a reaction diffusion equation to simulate a wave propagation in a 2D duct containing a bend (Pocci et al., 2010). They show how sharp bends naturally block the wave propagation. Above a critical radius blocking can be achieved by changing the system parameters, suggesting that adapted therapeutic agents could stop migraine aura. In a personalized approach to migraine aura treatment, Dahlem and his collaborators propose to use the Gaussian curvature of the cortex (computable from MRI data) to identify potential targets for neuromodulation (Dahlem et al., 2015). Applying a generic reaction diffusion model they highlight the local effects of the curvature on a simple 2D geometry with a bump, and they track the propagation path of a stable wave segment on a portion of the primary visual cortex obtained from an MRI scan.

Although these recent studies of CSD focused on personalized details of the brain geometry, still a realistic neural modeling perspective that approaches the whole cortex is lacking. Our goal is to study the wave propagation on a whole 3D individual geometry to identify symmetries and asymmetries in its behavior and analyze regions with respect to their potential to play a key role in CSD episodes.

In particular, we formulate a mathematical model of distributed neural excitability, and simulate the propagation of depolarization waves on an individual cortical geometry reconstructed from magnetic resonance imaging (MRI). The neural activity is described by a modification of the Rogers McCulloch variant of the FitzHugh-Nagumo model (Fitzhugh, 1961; Rogers and McCulloch, 1994) for excitable cells, and a diffusion term is added to account for the wave propagation. In order to localize the brain regions affecting propagation, we consider the Brodmann anatomical atlas (Brodmann, 2006). In each simulation, the wave propagation originates in one of the regions in the Brodmann atlas and we measure the arrival times to all the remaining regions. The data obtained from these simulations are post-processed to derive computable Quantities of Interest (QoI) that identify symmetries and asymmetries in the propagation of the depolarization wave.

## 2. Materials and methods

### 2.1. MRI acquisition

We are making use of one dataset already acquired and published in Diez et al. (2015). The work was approved by the Ethics Committee at the Cruces University Hospital and consequently all the methods were carried out in accordance to approved guidelines. We have considered here one data set corresponding to one healthy subject, male, age 28, and reanalyzed here by simulating a computational model of CSD on the cortical mesh. Data was acquired with a Philips Achieva 1.5T Nova scanner. The cortical mesh was obtained from a high-resolution anatomical MRI, acquired using a T1-weighted 3D sequence with the following parameters: *TR* = 7.482 ms, *TE* = 3.425 ms; parallel imaging (SENSE) acceleration factor = 1.5; acquisition matrix size = 256 x 256; FOV = 26 cm; slice thickness = 1.1 mm; 170 contiguous sections.

### 2.2. Realistic cortical geometry

The cortical geometry we used in this study has been reconstructed from a MRI scan with FreeSurfer image analysis suite, which is documented and freely available for download online at http://surfer.nmr.mgh.harvard.edu/. This processing includes removal of non-brain tissue using a hybrid watershed/surface deformation procedure (Ségonne et al., 2004), automated Talairach transformation, intensity normalization, tessellation of the gray/white matter boundary, automated topology correction, and surface deformation following intensity gradients to optimally place the gray/white and gray/cerebrospinal fluid borders at the location where the greatest shift in intensity defines the transition to the other tissue class. For further details, see Fischl (2012) and references therein.

### 2.3. Brain regions of interest

In order to analyze the impact of the geometry on the depolarization wave propagation, we consider a subdivision of the brain cortex into different regions of interest (ROIs). In particular, we base our study on the anatomical subdivision of each hemisphere into 34 ROIs, which is a generalized version of the Brodmann atlas (Brodmann, 2006) (included in the MRIcro software http://www.mricro.com). Such subdivision is available as online Supplementary Material to this paper. Another more general classification of the cerebral cortex is based on a coarser topographical conventional subdivision into six lobes: the medial and lateral temporal lobes, occipital lobe, parietal lobe, frontal lobe and cingulate cortex. Notice that although this classification is purely anatomical, it is well-known that different lobes are associated to different brain functions (Kandel et al., 2000).

### 2.4. Neuron modeling: A computationally efficient Fitzhugh-Nagumo distributed model

A key property of neural cells is to produce an action potential (AP). It consists in a sudden variation in the transmembrane potential, called spike, followed by a recovering of the resting condition through a refractory period, during which the cell cannot be excited. The Izhikevich's model is a classical 2 variable model describing the spiking behavior of cortical neurons (Izhikevich, 2007). Its drawback is the lack of autonomous behavior, as it needs a manual after-spike resetting of the variables. Such drawback is overcome by the model proposed in Cressman et al. (2009) that features self-sustained spiking and recovery cycles. In this model the firing rate can be modulated by acting on a parameter *k*_0, ∞_ which represents the concentration of potassium [K^+^] in the largest nearby reservoir. Modulating the firing rate allows to reproduce both resting and excited neuronal dynamics. In agreement with previous computational studies (Cortes et al., 2012), we consider neurons at rest to have a background firing rate of 4 Hz while excited neurons fire with an average frequency of 64 Hz.

With regard to computational considerations, first, because the time scale of the neural electrical activity is given in milliseconds, to simulate a frequency of 64 Hz with a detailed neuronal model would prompt the use of an extremely small time step (0.1 ms). But, with respect the time scale of the wave propagation at the cortical level (around 20 min), this makes the simulations to be computationally very expensive. In order to avoid unnecessarily heavy computations, we therefore describe the neuronal activity by deriving a slow variables model for the firing rate, where the state variable *u*(*x, t*) represents the average firing rate of neurons at location *x* and time *t* (in seconds). Such model can thus be locally considered a temporal mean field model with respect to the finer scale of the action potential. The model is inspired by the Rogers-McCulloch variant of the 2 variables FitzHugh-Nagumo model for excitable media (Fitzhugh, 1961). Such variant describes the all-or-nothing response of a single excited cell in a simplified manner (Rogers and McCulloch, 1994), and it exhibits autonomous behavior, ensuring the robustness of its numerical simulation. We modify the Rogers-McCulloch model to adapt the resting value (4 Hz), the spike value (64 Hz) and the plateau length in order to match the duration of the neuron excitation after the passage of the CSD (around 10 min, Porooshani et al., 2004).

Finally, a diffusion term accounts for the spatial propagation of the excitation. Thus, the complete model reads

(1) $$\begin{array}{rcl} \frac{\partial u}{\partial t} & = & -I\left(u,w\right)+\text{div}\left(D\nabla u\right) \end{array}$$

(2) $$\begin{array}{rcl} I\left(u,w\right) & = & G\left(u-u_{0}\right)\left(1-\frac{u}{u_{th}}\right)\left(1-\frac{u}{u_{p}}\right)+\eta_{1}\left(u-u_{0}\right)w \end{array}$$

(3) $$\begin{array}{rcl} \frac{\partial w}{\partial t} & = & \eta_{2}\left(u-u_{0}-\eta_{3}w\right), \end{array}$$

where *u*(*t*) is the firing rate at time *t* ≥ 0, and *w*(*t*) is the recovery variable, *u*_*th*_ and *u*_*p*_ are threshold and peak values for *u*, *u*_0_ is the background firing rate and *D* ∈ ℝ^3 × 3^ is the diffusion tensor (possibly anisotropic), while η_1_, η_2_, η_3_ and *G* are parameters, whose values are given in Table 1. The above equation is a coupled PDE-ODE system for all points (*t, x*) in the computational domain (0, *T*) × Ω, Ω ⊂ ℝ^3^. To have a mathematically well posed problem, initial conditions *u*(0, *x*), *w*(0, *x*) in Ω, and boundary conditions on ∂Ω have to be imposed. If the computational domain is a 2D surface Σ ⊂ ℝ^3^ the classical divergence and gradient operators are replaced by their tangential counterparts div_Σ_ and ∇_Σ_. Boundary conditions are not necessary if the surface Σ is closed, as in the case of the reconstructed cortical geometry we described in Section 2.2.

**Table 1:** **Model parameters**.

**Parameter**	**Description**	**Value**
*G*		1.6
*u*_0_	Resting value	4
*u*_*th*_	Threshold parameter	11.8
*u*_*p*_	Peak value	64
η_1_, η_2_, η_3_		2.9227, 2.e-4, 60

### 2.5. Numerical implementation

The computational grid consists of a triangulation of the reconstructed cortex. The lack of axial symmetry in the brain results in a mesh with 140.208 nodes and 280.412 triangles for the left hemisphere, and a mesh with 139.953 nodes and 279.902 triangles for the right hemisphere. Problem (1)–(3) is discretized in space by ℙ_1_ finite elements, while the time derivative is approximated by finite differences. Let *t*^*n*^ = *n*Δ*t*, for *n* = 0, .., *N* = *T*∕Δ*t*, be a discretization of the time interval (0, *T*): we denote with *u*^*n*^ and *w*^*n*^ the approximation of *u* and *w* at time *t*^*n*^. We use an implicit-explicit (IMEX) scheme to advance from *t*^*n*^ to *t*^*n*+1^: the recovery variable *w*^*n*+1^ is updated by solving explicitly (after linearization around *u*^*n*^) Equation (3) in (0, Δ*t*) and plugged into the expression of *I*(*u, w*) for the computation of *u*^*n*+1^. The overall procedure can be summarized as follows

Given *u*^*n*^ and *w*^*n*^,

(4) $$\begin{array}{l} \text{update: }w^{n+1}=\;\frac{u^{n}-u_{0}}{\eta_{3}}+\left(w^{n}-\frac{u^{n}-u_{0}}{\eta_{3}}\right)\exp\left(-\eta_{2}\eta_{3}\Delta t\right)\\ \text{update: }I^{n+1}=\;I(u^{n},w^{n+1})\\ \text{ solve: }Au^{n+1}=\;Mu^{n}-\Delta tMI^{n+1} \end{array}$$

where *A*: = *M* + Δ*tS*, where *M* and *S* are the standard finite elements mass and stiffness matrices (Quarteroni and Valli, 1997). A detailed description of the derivation of this system and an explicit formulation of the matrices *M* and *S* is available as Supplementary Material.

### 2.6. Simulation protocol

The numerical simulations of Equations (1)–(3) are performed with a self-developed code in Matlab (MathWorks Inc., Natick, MA) with a uniform time step of Δ*t* = 0.01 min. For every time step we solve the linear system in (5) with the conjugate gradient method, preconditioned by an incomplete Cholesky factorization (Saad, 2003). The diffusion tensor *D* = δId is isotropic with δ = 0.7174mm^2^s^−1^. This conductivity coefficient has been tuned to ensure that a wave is actually propagating across the cortex, at a velocity comparable with the one of the CSD. In general, smaller conductivities still trigger a propagating wave front. However, below a given threshold (δ ≤ 0.014mm^2^s^−1^) the refractory period is no longer a unidirectional restriction due to the slow propagation speed, allowing new patterns to emerge and spread across the cortex.

CSD is known to originate from the visual cortex, but to gain deeper insight in the way the geometry shapes the propagation, we simulate, in both hemispheres, the spread of excitation waves between all the regions of the anatomical classification. In each simulation, we consider as initial condition one fully depolarized region out of the 34 in the anatomical subdivision. Namely, we set *u*(0, *x*) = *u*_*p*_ for all *x* in the initially excited region, *u*(0, *x*) = *u*_0_ for all the remaining grid points and an initial uniform resting condition for the gating variable, *w*(0, *x*) = 0. Each simulation is run until all remaining regions have been fully activated. The arrival times of the depolarization wave in the remaining 33 regions are recorded. The only compartment that is not considered as initially activated is the corpus callosum as it constitutes the intersection between the two hemispheres and obeys different rules for diffusion. In particular, high anisotropy values of white-matter tracts, as revealed by tensor diffusion imaging, yield a much larger diffusion within corpus callosum with respect to other cortical areas. Our modeling strategy is indirectly accounting for this aspect: the mesh geometry within this region is much flatter in comparison with the other areas and, as a consequence, the simulated CSD propagation is faster.

**Figure 1:**
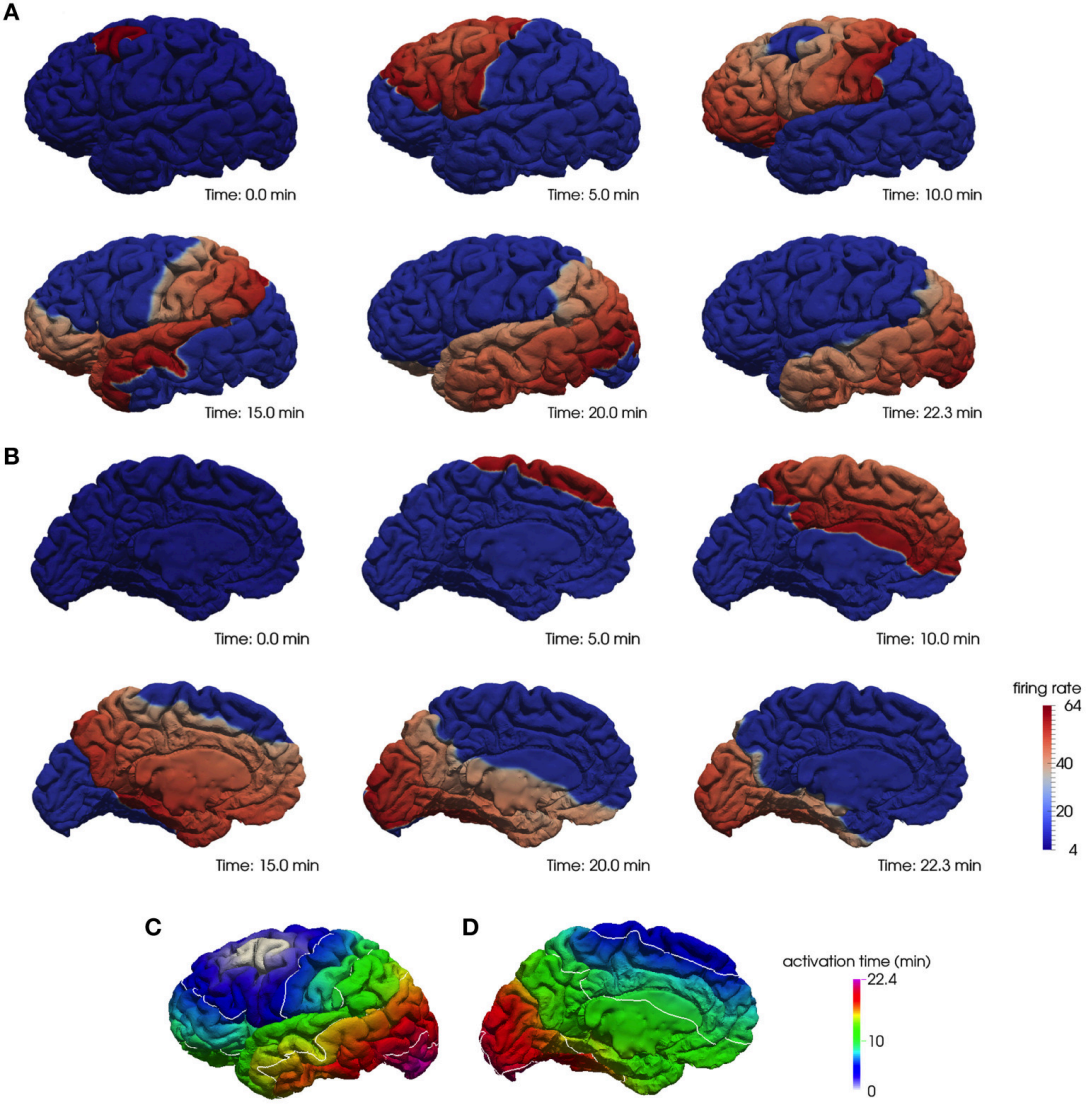
**A computational model of CSD on a realistic cortical geometry**. The progression of a depolarization wave starting in the caudal middle frontal region on the lateral **(A)** and medial **(B)** surface of the left hemisphere and the corresponding activation times, respectively, **(C,D)**.

As an illustration, we show in Figure 1 the progression of the depolarization wave starting from the caudal middle frontal region and spreading across the whole cortex for the lateral (A) and medial (B) surface of the left hemisphere. In Figures 1C,D we plot the corresponding activation times of the whole hemisphere.

### 2.7. Quantities of interest

Using the data obtained from the 34 numerical simulations per hemisphere, each one starting in a different Brodmann area, we can introduce different ways to assess symmetry and asymmetry in the propagation, with the aim of identifying regions featuring a peculiar propagation behavior.

In any simulation, one region is initially activated, and we record two values for each of the 33 remaining regions: the minimum and the maximum activation times. The minimum activation time is the moment when the first point of the region at hand gets excited, while the maximum activation time is the moment at which the last point of the region gets excited. The minimum activation time for a given arrival region does not depend on its shape or size, but only on the initially activated region and the portion of the cortex traveled by the wave between the two areas. On the other hand, the maximum activation time is also related to the shape and area of the arrival region. The two recorded quantities allow to assess different aspects of the propagation. The minimum activation time for a region provides information about the propagation behavior of its neighborhood. The maximum activation time is also accounting for the effect of the region's geometry on the propagation.

These quantities are collected into four 34 × 34 matrices, that we denote by *L*_*min*_, *L*_*max*_, *R*_*min*_, and *R*_*max*_, where *L* and *R* refer to the left and right hemisphere, respectively. In all of the above matrices, rows represent the starting region of the wave propagation, while columns the arrival region: as an example, the (*i, j*)-th element of *L*_*min*_ represents the arrival time in region *j* of a wave originated in region *i*.

**Figure 2:**
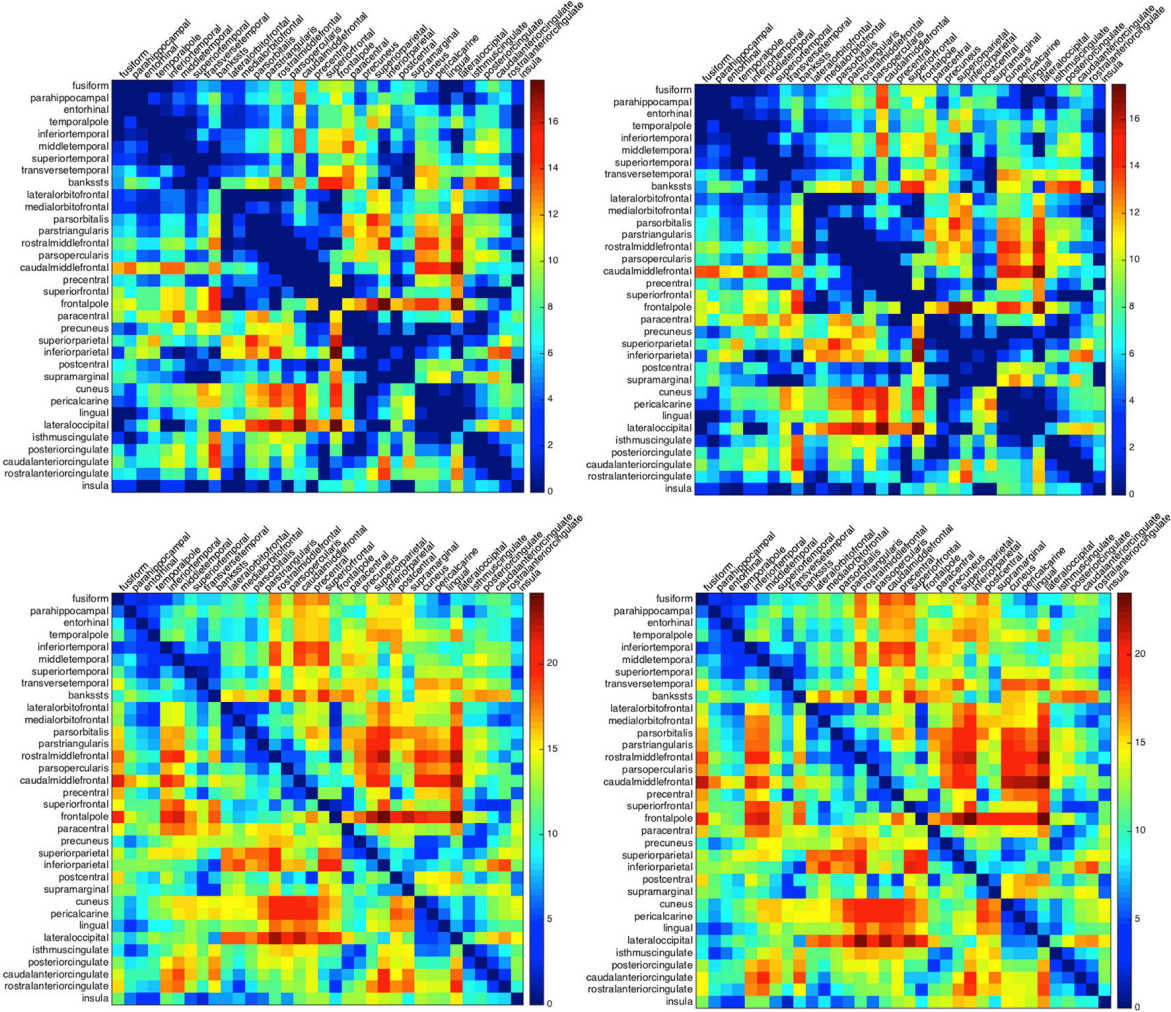
**Minimum and maximum activation times for CSD starting at one of the Brodmann areas and arriving to another**. Activation times for the left (left column) and right hemisphere (right column): minimum (top row) and maximum activation time (bottom row). Rows indicate the initial regions for the depolarization while columns indicate the arrival regions. The abbreviations on the left hand side denote the lobes: m.t., medial temporal lobe; l.t., lateral temporal lobe; f.l., frontal lobe; p.l., parietal lobe; o.l., occipital lobe; and c.c., cingulate cortex.

The ordering of the regions in building such matrices plays a crucial role in the clustering of the results. Different sorting choices, like the clustering of all regions with respect to their centroids distances or the clustering with respect to the activation times, lead to different types of clustering. To emphasize the spatial connection between the regions, we chose to rearrange their ordering according to their affiliation to lobes. Regions belonging to one lobe are then clustered according to the mutual distance of their centroids in the Euclidean norm. The minimum and maximum activation times for the left as well as for the right hemisphere are given in Figure 2.

Whatever the ordering of the regions, a lack of symmetry in the matrices *L*_*min*_ and *R*_*min*_ is a first clear indicator that the geometry plays a role in shaping the propagation: if the cortex had been spherical, an isotropic conductivity coefficient would have resulted in both matrices being symmetric.

#### 2.7.1. Correlation between activation times and distances

Despite the complex geometry of the cortex, a direct relation between the propagation time of wavefronts and the distance traveled is expected. However, to further assess differences between the left and right hemisphere, we explicitly relate activation times with distances from the initially activated region. We consider the Euclidean distance between the centroids of two regions as a proxy of the distance traveled by the wavefront.

The correlation between the distance of the centroids of the different regions and the different activation times can be assessed by the Pearson product-moment correlation coefficient, a measure of the linear correlation between two variables X and Y. The Pearson test returns values between +1 and -1 inclusive, where 1 is total positive correlation, 0 is no correlation, and -1 is total negative correlation. For a given sample of paired data (_*x*_*i*_, *y*_*i*_)*i* = 1, …, *n*_ the Pearson correlation coefficient is defined as

(5) $$\begin{array}{l} r=\frac{\sum_{i=1}^{n}\left(x_{i}-\bar{x}\right)\left(y_{i}-ȳ\right)}{\sqrt{\sum_{i=1}^{n}{\left(x_{i}-\bar{x}\right)}^{2}}\sqrt{\sum_{i=1}^{n}{\left(y_{i}-ȳ\right)}^{2}}} \end{array}$$

with $\bar{x}=\frac{1}{n}\overset{n}{\underset{i=1}{\sum }}x_{i}$ and $ȳ=\frac{1}{n}\overset{n}{\underset{i=1}{\sum }}y_{i}$.

#### 2.7.2. Assessment of bilateral asymmetry: The global asymmetry index

Most of the anatomical areas are present in the brain bilaterally. To assess propagation differences between brain hemispheres, we analyze how an excitation wave propagates differently in the two hemispheres. Indeed, comparing propagation speed in the different directions provides substantial information about the global geometry and how easily depolarization waves propagate through certain areas. The minimum activation time matrices *L*_*min*_ and *R*_*min*_ are the least dependent on the size and shape of the regions, and provide valuable information about the propagation in the neighborhood of all regions. As a consequence, these matrices are natural candidates to assess global symmetry properties on both hemispheres. First we focus on propagation asymmetry within each hemisphere, and we consider the time difference for a depolarization wave to travel back and forth between any two regions. Such difference is given by the matrices

(6) $$\begin{array}{l} L=L_{min}-L_{min}^{T}\;\text{and}\;R=R_{min}-R_{min}^{T}, \end{array}$$

where $L_{min}^{T}$ and $R_{min}^{T}$ denote the transposes of matrices *L*_*min*_ and *R*_*min*_. Each entry *L*_*ij*_ (respectively, *R*_*ij*_) expresses the difference in arrival time between a wave traveling from region *i* to region *j* of the left (respectively, right) hemisphere, and a wave traveling from region *j* to region *i*. If the propagation of the excitation wave is symmetric in any hemisphere, the corresponding matrix *L* or *R* vanishes. The absolute values of the entries of *L* and *R* provide a global measure for the asymmetry in the geometry between two different regions. As *L* and *R* are skew-symmetric matrices with zero diagonal, we can display their absolute values in a single matrix without loss of information (Figure 4).

To quantify the lack of symmetry in the propagation, we consider the normalized difference matrices $\hat{L}$ and $\hat{R}$, whose (*i, j*)-th entries are defined as

(7) $$\begin{array}{r} {\hat{L}}_{ij}=\{\begin{array}{ccc} \begin{array}{c} \;L_{ij}\;\text{for}\;i=j\\ \frac{L_{ij}}{{\left(L_{min}\right)}_{ij}}\text{for}\;i\neq j \end{array} & \;{\hat{R}}_{ij} & \{\begin{array}{c} \;R_{ij}\;\text{for}\;i=j\\ \frac{R_{ij}}{{\left(R_{min}\right)}_{ij}}\;\text{for}\;i\neq j. \end{array} \end{array}\; \end{array}$$

We denote by ${\bar{l}}_{j}$ and ${\bar{r}}_{j}$ the mean of the *j*-th column of matrix $\hat{L}$ and $\hat{R}$, respectively. For each region *j*, positive values of *l*_*j*_ (or *r*_*j*_) indicate that depolarization waves moving away from the region are faster than depolarization waves approaching that region. A global asymmetry index, for each region *j*, is then obtained by taking the sign of the quantities ${\bar{l}}_{j}$ and ${\bar{r}}_{j}$

(8) $$\begin{array}{r} A_{j}^{L}=sign\left(l_{j}\right)\;\text{and}\;A_{j}^{R}=sign\left(r_{j}\right). \end{array}$$

#### 2.7.3. Local residential time of the depolarization wave: The retention index

For a given region, the time the depolarization wavefront needs to sweep the whole region provides important information about its shape, size, and geometrical regularity. To assess possible differences in the local behavior between the left and the right hemisphere, we introduce the residence time as the time elapsed between the depolarization of the first and the last point in a region. The residence time depends not only on the shape and the size of a region, but also on the direction from which the depolarization wave is coming. An elongated region, for instance, would feature very different residence time if the incoming wave is entering the region along its short or its long axis. By considering a different initially activated region per simulation, all possible incoming directions are taken into account. We thus define the depolarization residence matrices by considering the difference between the maximum and minimum activation times, as

(9) $$\begin{array}{l} D^{L}=L_{max}-L_{min}\;D^{R}=R_{max}-R_{min}. \end{array}$$

Again, rows represent the starting region of the wave propagation, while columns the difference between activation times: the (*i, j*)-th element of D^*L*^ and D^*R*^ represents the residence time in region *j* of a wavefront originates from region *i*. Hence, the *j*-th column of D^*L*^ gives the residence times for the *j*-th region in the left hemisphere for all initially excited region, while the *j*-th column of D^*R*^ provides the same information for the corresponding *j*-th region in the right hemisphere. The behavioral symmetry (or lack of) between corresponding regions of the two hemispheres can thus be assessed by comparing the average residence time (both mean and median) from the columns of D^*L*^ and D^*R*^.

By summing up the entries of each column we obtain, for any given region, a global measure for the retention of the depolarization wave that is independent of the initial condition, thus implicitly taking into account the region's shape. For both hemispheres we introduce the retention index, that measures the overall time in which the depolarization wave stays in region *j*, and is defined as

(10) $$\begin{array}{l} R_{j}^{L}=\overset{34}{\underset{i=1}{\sum }}D_{ij}^{L},\;R_{j}^{R}=\overset{34}{\underset{i=1}{\sum }}D_{ij}^{R}. \end{array}$$

A direct relationship between the residence time of the depolarization wave in a given region and the area of the region itself is expected. We assess it by considering the datasets

(11) $$\begin{array}{l} {\left(s_{i},R_{i}^{L}\right)}_{i=1,\ldots ,34},\;\text{and}\;{\left(s_{i},R_{i}^{R}\right)}_{i=1,\ldots ,34}, \end{array}$$

where *s*_*i*_ describes the surface of region *i* and R_*i*_ the corresponding retention index. Since we focus on the identification of symmetries (and lack of them) we are particularly interested in regions whose propagative behavior deviates significantly from the others. A natural choice to detect regions of unusual behavior, is to look for outliers in the database. The standard method for multivariate outlier detection is the Mahalonobis distance. For an *n* × *p* dimensional set of data ${\left(x_{1},\ldots ,x_{n}\right)}^{T}$ with the *i*-th observation defined as *x*_*i*_ = (*x*_*i*1_, …, *x*_*ip*_) the Mahalonobis distance is defined as

(12) $$\begin{array}{l} MD_{i}=\sqrt{{\left(x_{i}-\mu \right)}^{T}S^{-1}\left(x_{i}-\mu \right)}\text{ for }i=1,\ldots ,n \end{array}$$

where μ is the estimated mean and *S* the estimated covariance matrix. For multivariate normally distributed data the values are approximately χ^2^-distributed with *p* degrees of freedom. Multivariate outliers can now be defined as observations having a large Mahalonobis distance. Thus, a quantile of the $\chi_{p}^{2}$ distribution, e.g., the 97.5% quantile, can be considered. A drawback of the Mahalonobis distance is that it makes use of the classical estimators for mean and covariance, which can be highly affected by outlying values. In order to obtain more reliable results for the data analysis, more robust estimators are required. The minimum covariance determinant (MCD) estimator is most frequently used and can be computed with a fast algorithm (Rousseeuw and Van Driessen, 1999). Using robust estimators of location and scatter in Equation (12) we obtain the so called robust distance (RD) (Rousseeuw and Van Zomeren, 1990, 1991).

## 3. Results

### 3.1. Correlation between activation times and distances

We first correlate the activation times with the euclidean distance between the centroids of the regions. The latter is used as a proxy for the actual distance traveled by the depolarization wave. We create 4 datasets from the off-diagonal entries of matrices *L*_*min*_, *L*_*max*_, *R*_*min*_, and *R*_*max*_. Diagonal elements of these matrices as well as the corresponding euclidean distances vanish, since the starting and arrival points coincide. Thus, we can neglect diagonal entries in the definition of the datasets, each of which consist of 1122 pairs.

**Figure 3:**
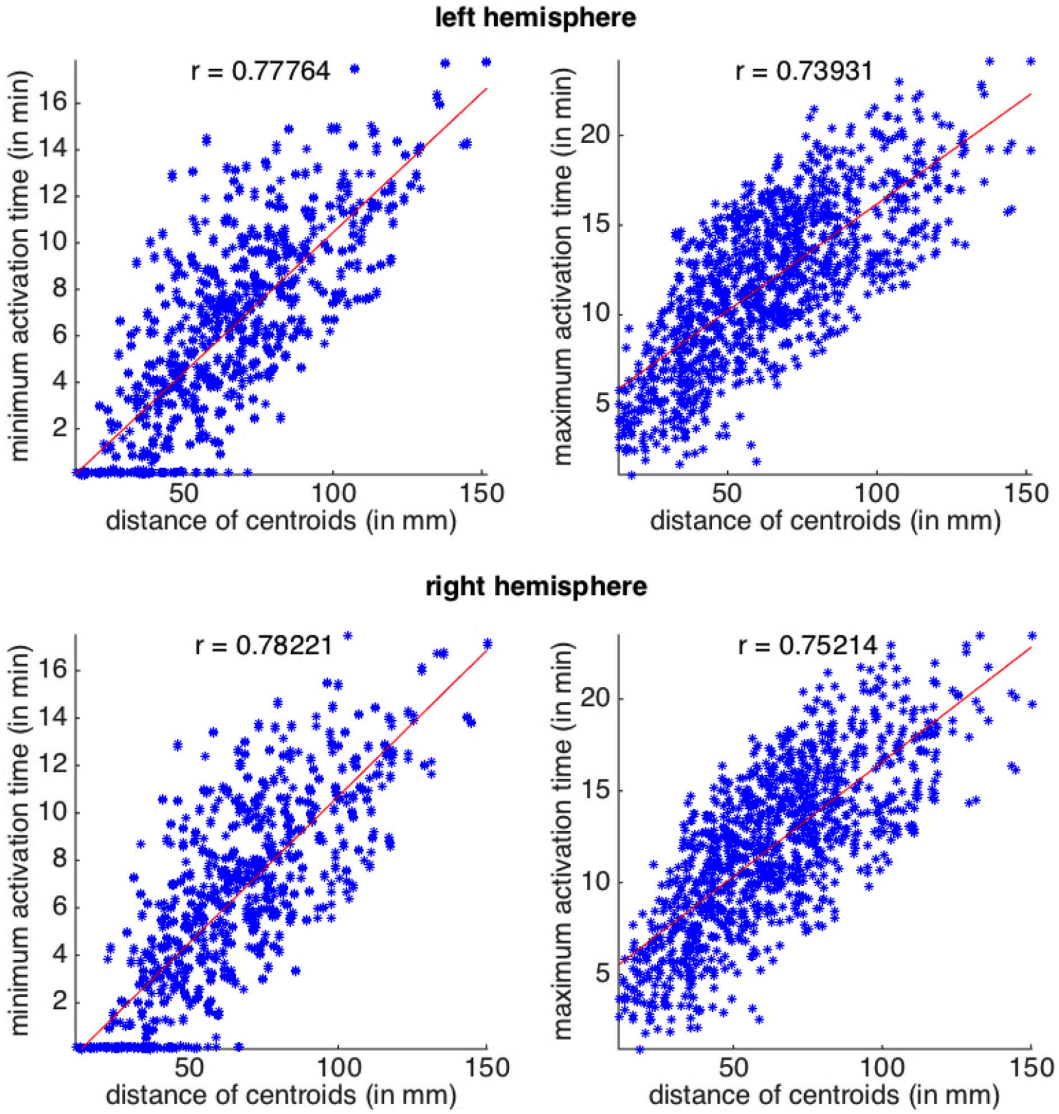
**Activation times (CSD starting in one area and arriving to another) as a function of Euclidean distance between the two areas**. Top row, left hemisphere and bottom row, right hemisphere. Scatterplots for the minimum and maximum activation time as a function of the Euclidean distance between region centroids. All *p*-values for the linear regression are below 10^−7^. The correlation coefficient, *r*, is also shown for each plot.

In Figure 3 we plot the four datasets, together with their linear regressions, all featuring *p*-values below 10^−7^. In all the plots, Pearson coefficients are high, revealing a positive correlation between the distance of the regions and the activation times. When comparing between the two hemispheres, we can observe a slightly stronger correlation between the activation times and the distances on the right hemisphere.

### 3.2. Assessment of bilateral asymmetry: The global asymmetry index

We then focus on the difference in propagation between hemispheres. To this aim we consider the matrices *L* and *R*, defined in (6), each of whose entries represent the time difference in a back-and-forth propagation between two regions. The absolute value of their entries provides a global estimator to which hemisphere features the most unsymmetric behavior. Since both matrices *L* and *R* are skew-symmetric with zero diagonal, their absolute values can be represented, without loss of information, by their lower or upper triangular, off-diagonal, portion, embedded in a single matrix *G*, whose entries are given by

(13) $$\begin{array}{r} G_{ij}=\left\{ \begin{array}{l} \left|L_{ij}\right|\;\text{for}\;i>j\\ 0\;\text{for}\;i=j\\ \left|R_{ij}\right|\;\text{for}\;i<j \end{array}\right. \end{array}$$

**Figure 4:**
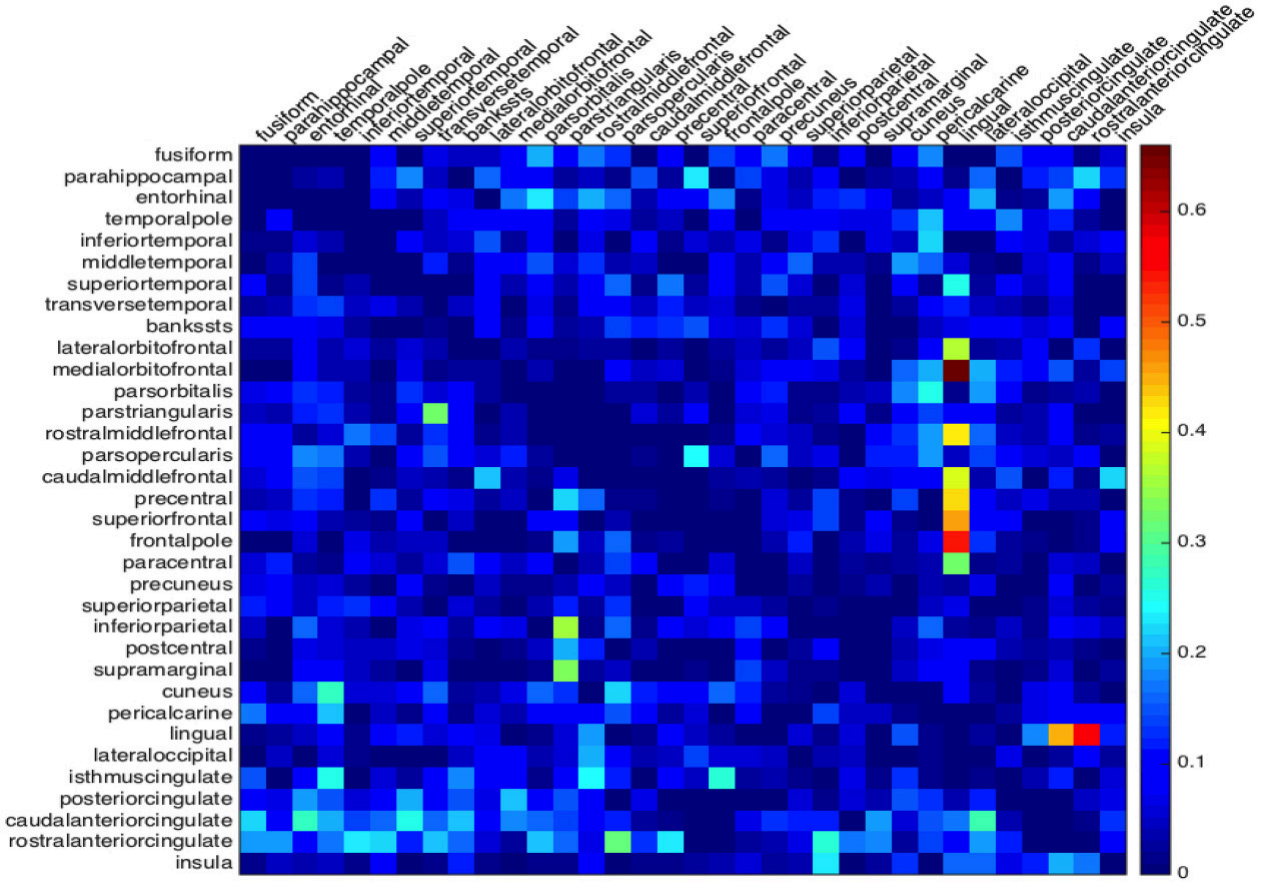
**Global asymmetry from the minimum activation time**. The absolute time difference of the depolarization wave to propagate between two regions back and forth. The lower triangle gives the values of the left hemisphere and the upper triangle the values for the right hemisphere.

The matrix *G* collects at once all the information on the lack of symmetry of both hemispheres, that can be compared by the single plot in Figure 4. For the cortical geometry at hand, we can observe a more significant asymmetry in the right hemisphere, in particular in the neighborhood of the lingual region.

**Figure 5:**
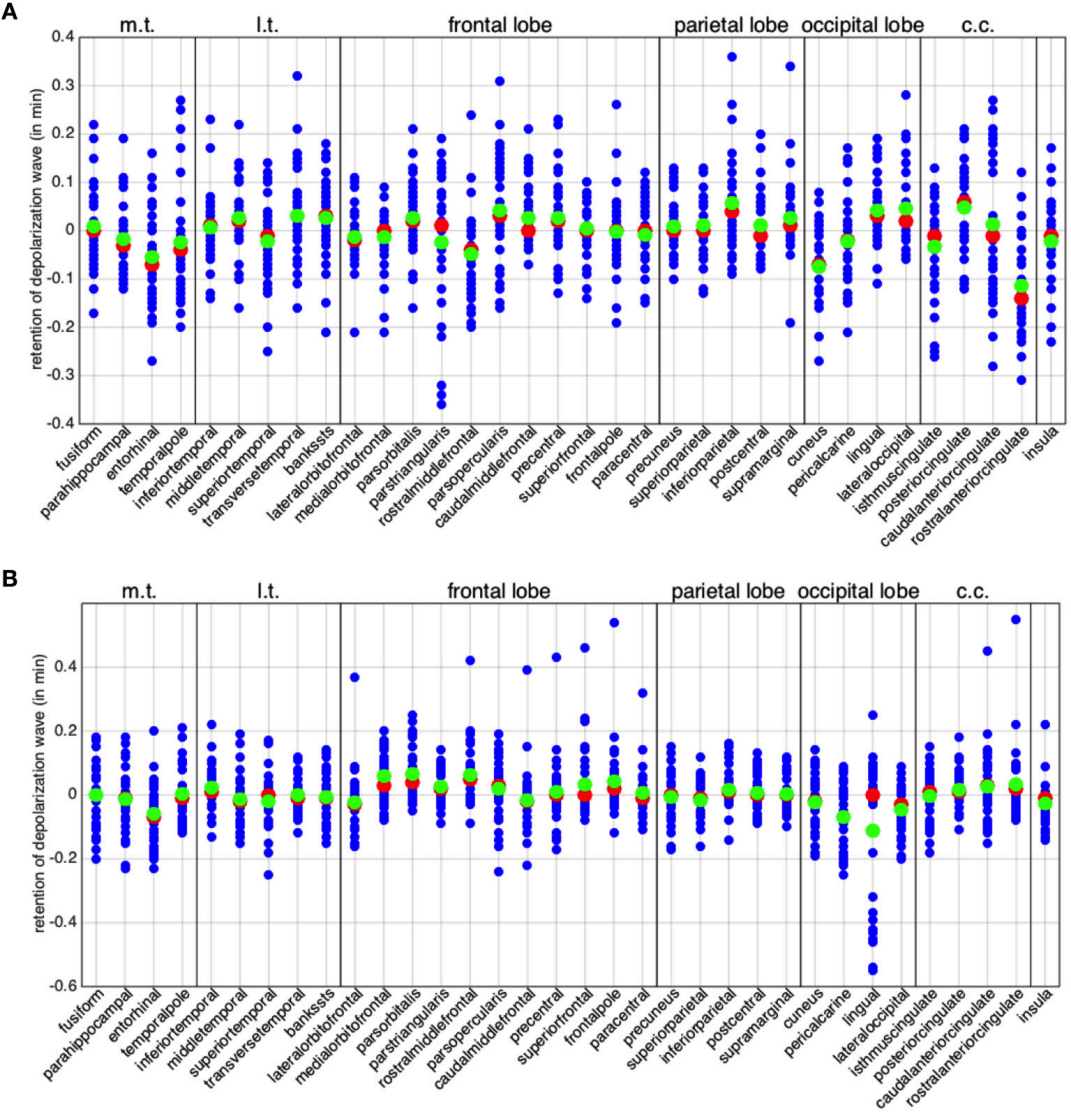
**Global asymmetry per brain region**. The differences in minimum activation time per brain region, i.e. the columns of matrices $\hat{L}$ and $\hat{R}$ defined in (Equation 7). **(A)** Left hemisphere. **(B)** Right hemisphere. The red points denote the medians and the green ones the arithmetic means. In all the plots, the abbreviation m.t. represents the medial temporal lobe, l.t. the lateral temporal lobe, and c.c. the cingulate cortex.

A more detailed assessment of the asymmetrical behavior of the two hemispheres is given in Figure 5, where we plot the columns of the matrices $\hat{L}$ and $\hat{R}$ defined in (7). Each column *l*_*j*_ (*r*_*j*_, respectively) represents the normalized difference in time propagation to and from the *j*-th region. Also in Figure 5 regions are clustered according to lobes. We highlight for all regions both the mean (in green) and the median (in red). We observe that, despite the means cluster around 0, several regions feature large variance, with differences up to 36 s in back-and-forth time propagation.

**Figure 6:**
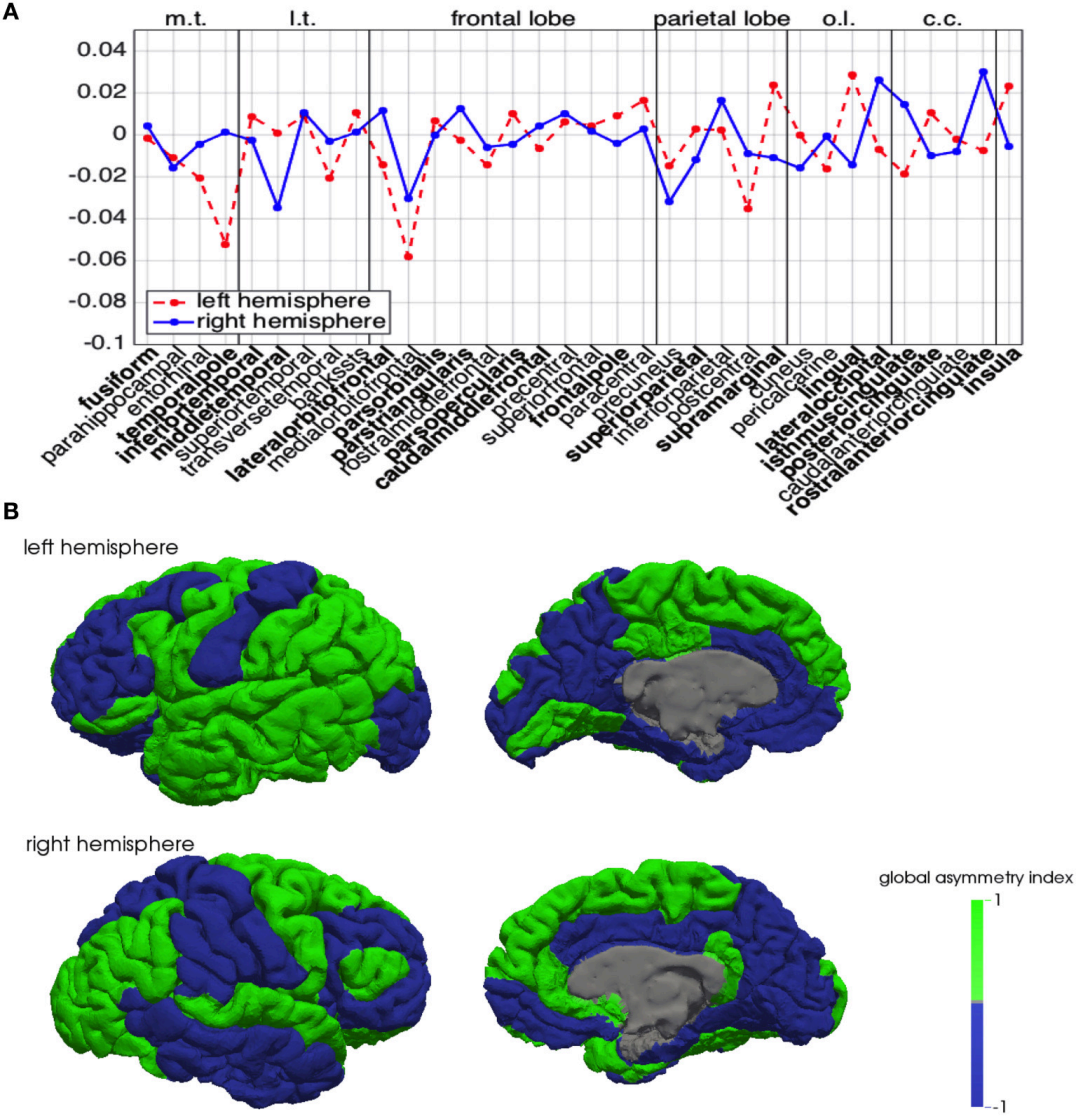
**Global asymmetry index across brain regions**. **(A)** Mean of the normalized differences $\hat{L}$ and $\hat{R}$ for the left (red dashed) and right hemisphere (blue). In the plot, m.t. denotes the medial temporal lobe, l.t. the lateral temporal lobe, o.l. the occipital lobe, and c.c. the cingulate cortex. The regions that exhibit different behavior in the two hemispheres are identified in boldface. **(B)** Brain spatial maps of the normalized global asymmetry index on the left (top row) and right hemispheres (bottom row).

A positive mean ${\bar{l}}_{j}$ (respectively, ${\bar{r}}_{j}$) implies that the corresponding region is, in general, behaving as a source (or a facilitator) for the propagation of a depolarization wave, while a negative mean implies that the region is behaving as a sink for the propagation. In Figure 6A, we summarize in the same plot the means ${\bar{l}}_{j}$ and ${\bar{r}}_{j}$. Half of the regions appear to behave in a similar manner in both hemispheres, while the other half shows opposite behavior in the two hemispheres. In addition, also among regions showing the same behavior, some feature significant differences between hemispheres, like the medialorbitofrontal, where ${\bar{l}}_{j}$ is twice ${\bar{r}}_{j}$.

In Figure 6B we plot the global asymmetry index introduced in (8), that visually highlights the behavioral differences between homologous regions in the two hemispheres.

### 3.3. Residence of the depolarization wave: The retention index

**Figure 7:**
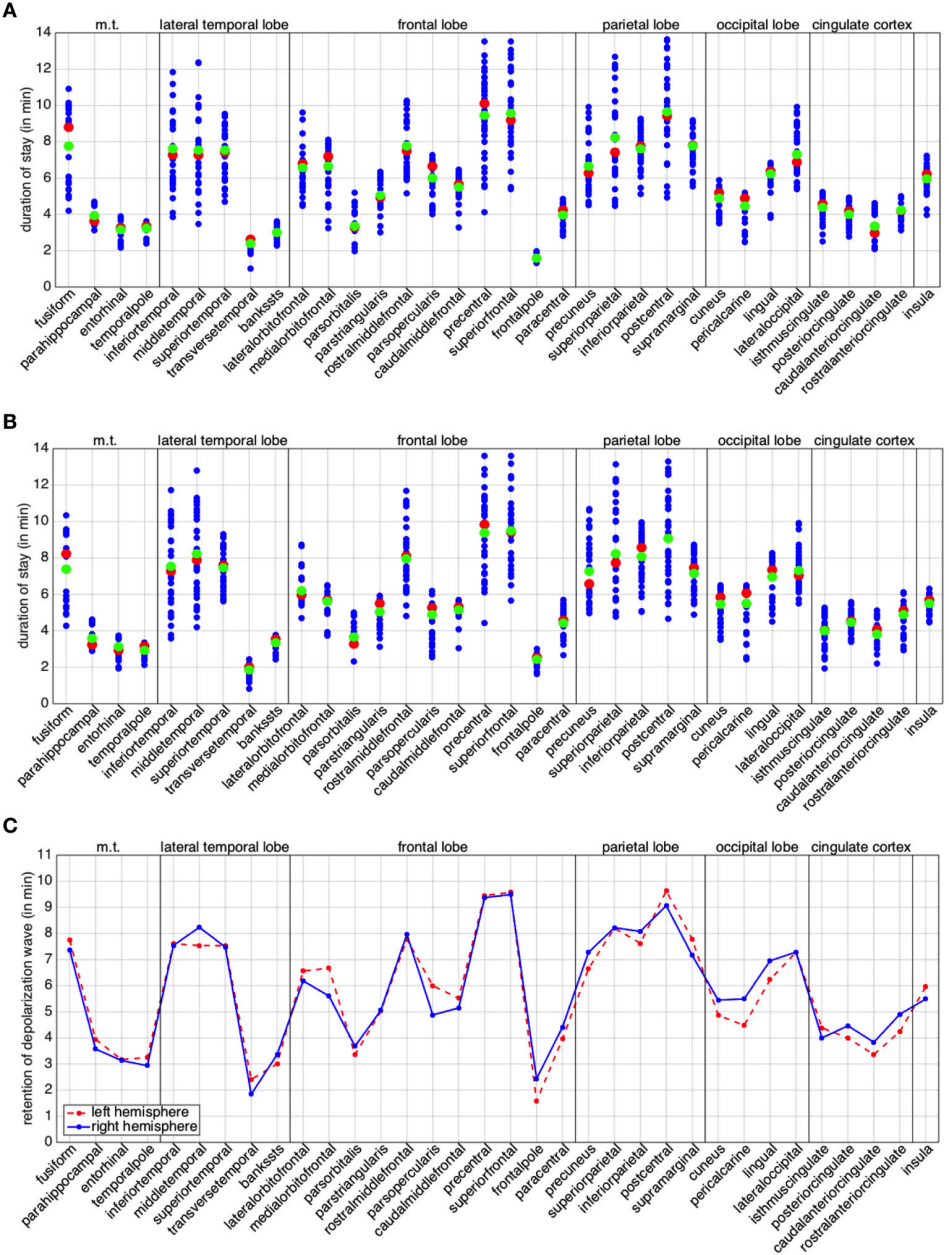
**Retention index per brain region**. Duration of stay of the depolarization wave for the regions of the left **(A)** and right **(B)** hemisphere. The red markers denote the median, the green ones the arithmetic mean. **(C)** Comparative plot of the mean for regions in the left (red dotted) and right (blue) hemisphere. In all the plots, the abbreviation m.t., denotes the medial temporal lobe; l.t., the lateral temporal lobe; c.c., cingulate cortex.

To conclude this study, we turn our attention to the individual behavior of the regions in the anatomical decomposition. For each region we plot the residence of the depolarization wave, given by the columns of matrices D^*L*^ and D^*R*^, defined in (9). We also compute median and arithmetic mean of the column entries for left and right hemisphere (Figures 7A,B). By construction, the diagonal elements of matrices D^*L*^ and D^*R*^ are zero and are once again neglected as they are not informative. Wide varieties in the residence depolarization time hint at elongated or very irregularly shaped regions, in terms of their curvature or basal area. We point out that the times presented in Figure 7 (A through C) do not actually represent the duration of the excitation of the whole regions. The excitation period for the cortical cells after the passage of the CSD is of the order of 10 min. As a consequence, for elongated regions, repolarization can occur before the whole region has been activated. From Figures 7A,B we can observe that, for the cortical geometry at hand, 8 regions in the left hemisphere and 9 in the right hemisphere are characterized by such a feature.

In order to study the behavior of the different hemispheres, we compare the means of the corresponding regions (Figure 7C). In our case study we can observe a higher similarity in the behavior between the two hemispheres with respect to the back and forth propagation studied in the previous section. A few regions exhibit appreciably different behavior, likely the consequence of the lack of anatomical symmetry between the two hemispheres: such regions are the middletemporal, the medialorbitfrontal, the parsopercularis, the cuneus, the pericalcarine, the lingual, the isthmuscingulate, the posteriorcingulate, the caudalanteriorcingulate and the rostralanteriorcingulate. From Figure 7C, we can observe that, for the cortical geometry at hand, the major differences are localized in the occipital lobe and in the cingulate cortex.

**Figure 8:**
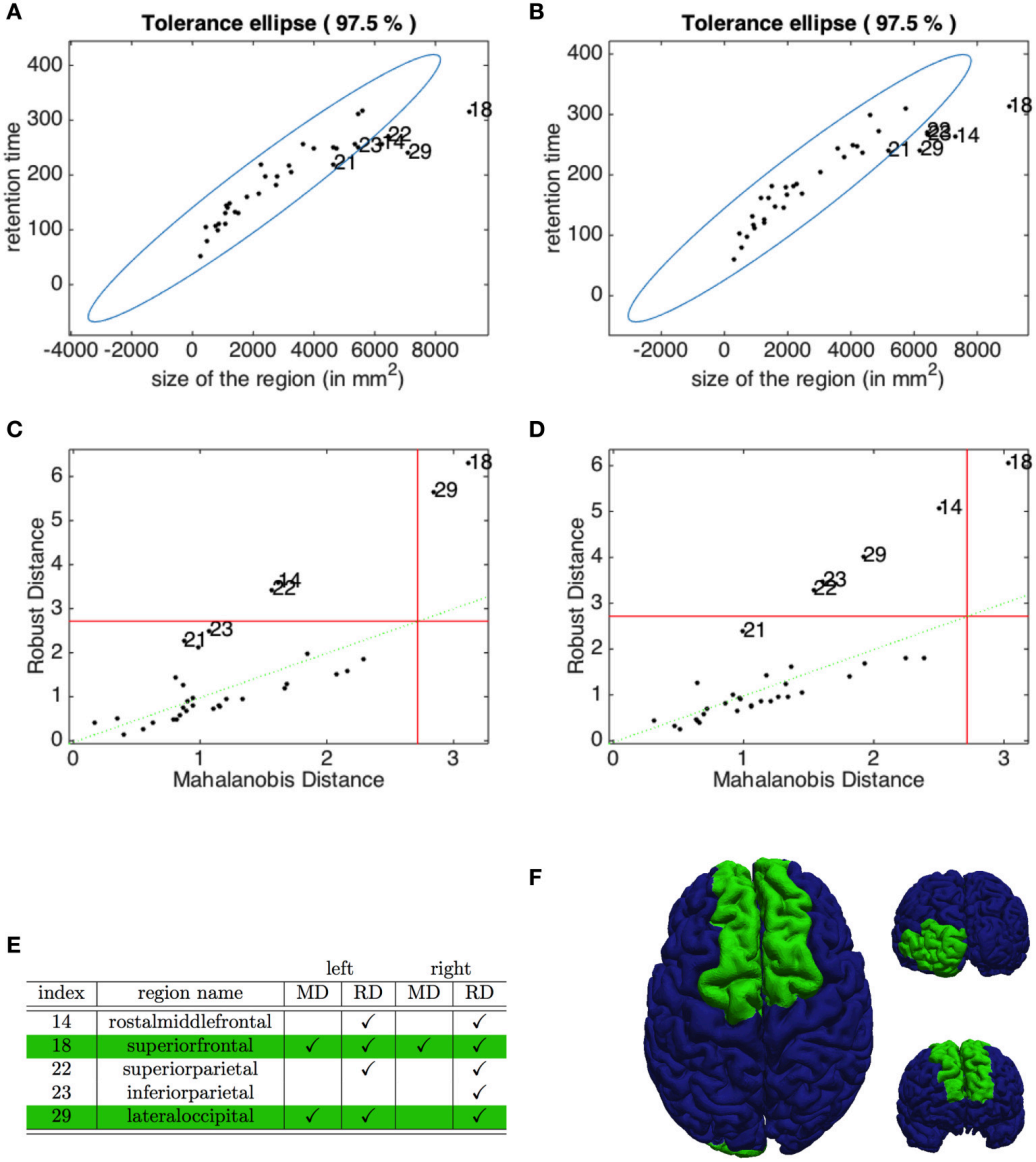
**Identification of outliers in the relation between retention index and region size**. The tolerance ellipse of the 97.5% quantile for the robust distance for the data form the left **(A)** and the right hemisphere **(B)**. Numbers refer to the index of the datasets. The distance-distance plot of the Mahalonobis distance and the robust distance for the left **(C)** and right hemisphere **(D)** where the red lines mark the 97.5% quantile. **(E)** The index and name of the outlying regions for the left and right hemisphere detected with the Mahalonobis distance (MD) and the robust distance (RD). The checkmarks indicate that this dataset was identified as an outlier by the corresponding method. **(F)** The superior, anterior and posterior view of the brain highlighting the regions detected as outliers by both MD and RD methods.

The Pearson correlation coefficients, introduced in (5), highlight in both hemispheres a clear positive correlation (*r* = 0.9087 for the left and *r* = 0.9103 for the right hemisphere) between the residence time of the depolarization wave and the size of the corresponding region. To identify the outliers, we plot in Figures 8A,B the robust tolerance ellipse describing the 97.5 % quantile for the datasets of the left and right hemisphere. In the whole Figure 8, numbers identify regions according to their ordering described in Section 2.7. The Mahalonobis distance and the robust distance are compared in the distance - distance plots in Figures 8C,D. In all graphs, the 97.5% quantile of the $\chi_{2}^{2}$-distribution is drawn as a threshold value. The detected outliers with respect to the two distances are identified both by their indexes and the corresponding region names, and comparatively collected in the table given in Figure 8E. The regions that are rated as outliers by both methods are identified on the cortex of our brain geometry at hand in Figure 8F.

## 4. Discussion

We have presented a computational model of CSD and studied how cortex geometry shapes propagation of CSD. The realistic geometry is provided by an individual subject-specific mesh reconstructed from high-resolution structural MRI. The computational CSD model is described by a PDE-ODE system resulting from a modification of the Rogers McCulloch variant of the FitzHugh-Nagumo model. The model features a slow dynamic variable, representing the firing rate of the neurons, and a recovery variable. The model is integrated by an IMEX (IMplicit/EXplicit) finite element scheme. Resorting to the Brodmann's atlas, we divided each brain's hemisphere in 34 different anatomical regions, and we identified suitable Quantities of Interest (QoIs) that can be computed by postprocessing the results from the simulation of the CSD propagation across the whole cortex.

We have studied propagation symmetries and asymmetries by identifying them both intra and inter-hemispherically. By introducing the global asymmetry index and the retention index (see Methods), we have found a clear asymmetry pattern emerging in both assessments. In particular, we observed that the propagation speed between two non-neighboring regions *i* and *j* is not the same when comparing CSD traveling in opposite directions. That is, the speed of a wave moving from *i* to *j* is different from the one of a wave moving from *j* to *i*, and this feature is common to both left and right brain hemispheres.

Neuroanatomical differences between the left and right sides of the brain are known to exist at various scales (Toga and Thompson, 2003); our work shows that asymmetrical evidences also occur for the propagation of waves of electrophysiological activity, most likely due to geometric effects (but might also be due to differences in brain circuits within left and right hemisphere). A recent work highlights the local influence of the curvature of two-dimensional surfaces on properties, such as nucleation and propagation of waves (Kneer et al., 2014); our results investigate this relation at the scale of a whole cortical geometry reconstructed from brain imaging.

Still, the cause of the propagation asymmetry is not completely clear yet and is the subject of our ongoing investigation. In particular, since geometry is expected to have a bigger impact on the propagation on a two-dimensional surface with respect to a three-dimensional structure, we plan to investigate the relation between the cortex curvature and the emerging asymmetries.

We have also identified brain regions whose behavior significantly differs from the other regions. In particular, some regions appear to trap the propagating action potentials for a longer time. These outliers in the relation between retention index and region sizes are strong candidates to identify areas that may play a key role in the CSD propagation (and possibly be able to stop it). Such information would be relevant to design therapies using stereotactic cortical neuromodulation, where target structures for modulation have to be carefully selected, see, e.g., Dahlem et al. (2015). Implementing an individualized computational model for CSD would improve the clinical effectiveness of these therapies.

In this paper we have modeled the diffusion tensor as isotropic, with all eigenvalues being equal to a constant δ. However, diffusion tensor imaging (DTI) provides, per a given voxel in the image, a more realistic (strongly anisotropic) tensor for the diffusion of water molecules across white-matter tracts. We expect that more personalized conductivity values, taking into account information from DTI data, can provide a better insight on the regions that are principally responsible for this lack of symmetrical behavior.

The model can benefit from the incorporation of other ingredients. An important limitation of our study is that the diffusion model does not account for long-range connections. Rather than adopting one of the many computational strategies to model such features within a circuit (for instance, by adding random edges between far-separated mesh nodes), we preferred to just focus on short-range connections associated to excitatory connectivity. In addition, we have not modeled cortical inhibition, which is a well-known mechanism for controlling propagation of neuronal excitability in cortical circuits. Incorporating inhibitory neurons and long-range connections within the diffusion model will be of special interest for future research on CSD modeling. Indeed, long-range inhibitory connections have been found in several sensory cortices where they play a key role in stabilizing strong increases of electrical activity. Notice that, the presence of inhibition, in addition to make the model more realistic, will scale the diffusion constants, making them several orders of magnitude higher in order to produce CSD propagating at the macroscale with a time duration of about 20 min. Moreover, the incorporation of short-time synaptic plasticity, including synaptic depression and facilitation (Markram and Tsodyks, 1996; Tsodyks and Markram, 1997) makes neural connectivity to be activity-dependent, adding new non-linearities into the model which might strongly affect the stability of CSD propagation (Cortes et al., 2013). Finally, the use of a more detailed neuronal model, instead of the firing rate slow dynamics at the basis of our analysis, would allow to study, at the cost of increased computational effort, the impact of channelopathies in favoring or countering the propagation of CSD.

To conclude, some words have to be said in relation to the disease. Based on the evidence that CSD has been proposed to be a neural correlate of aura migraine, we have presented a method that addresses dynamical features of CSD propagating on a realistic (subject-specific) cortical geometry. Computational models of migraine, in synergy with the analysis of altered processing of sensory stimuli (de Tommaso et al., 2014), not only provide further insight in this disease, but they are also fundamental in constructing new interventional approaches. The cortical data we used in this paper is coming from an healthy brain, and can provide a baseline for the QoIs that we identified. Whether the results shown here remain valid or not, and asymmetries are enhanced when simulations are run on a mesh coming from a patient suffering from aura migraine, is for us an obligated matter for future research. The occurrence of larger deviations from the baseline in unhealthy people, would make the QoIs introduced in this paper able to discriminate healthy from unhealthy patients, and, at the same time, able to identify subjects potentially susceptible of suffering from migraine aura. We expect further insight in this direction by the extensive application of our analysis to a statistically significant sample of patients.

### Conflict of interest statement

The authors declare that the research was conducted in the absence of any commercial or financial relationships that could be construed as a potential conflict of interest. The reviewer Dr Wang and handling Editor, Dr Wu declared their shared affiliation, and the handling Editor states that the process nevertheless met the standards of a fair and objective review.
