# Supplementary material for: Geometry Shapes Propagation: Assessing the Presence and Absence of Cortical Symmetries through a Computational Model of Cortical Spreading Depression
Source: Front Comput Neurosci. 2016 Feb 2;10:6. doi: 10.3389/fncom.2016.00006 (PMC4735361; doi:10.3389/fncom.2016.00006)
Supplement: Supplementary file 1 [file Presentation1.pdf]

Supplementary Material: Geometry shapes propagation: assessing the presence and absence of cortical symmetries through a computational model of cortical spreading depression

Julia M. Kroos*, Ibai Diez, Jesus M. Cortes, Sebastiano Stramaglia and Luca Gerardo-Giorda

*Correspondence:
Julia M. Kroos
BCAM – Basque Center for Applied Mathematics, Alameda de Mazarredo 14,
E48009 Bilbao, Basque Country – Spain
jkroos@bcamath.org

1 SUPPLEMENTARY MATERIAL

1.1 Numerical Approximation of the Distributed Model

For the time discretization of the model

$$\begin{aligned} \frac{\partial u}{\partial t} &= -I(u,w) + \mathrm{div}(D\nabla u) &(1)\\ I(u,w) &= G(u-u_0)\left(1-\frac{u}{u_{th}}\right)\left(1-\frac{u}{u_p}\right) + \eta_1(u-u_0)w &(2)\\ \frac{\partial w}{\partial t} &= \eta_2\left(u-u_0-\eta_3 w\right), &(3)\end{aligned}$$

we consider a uniform mesh in the time variable t and define $t^n = n\Delta t$ for $n = 0, \ldots, N$ with the time step $\Delta t = T/N$ and $t \in [0,T]$. In the following u^n denotes the discrete evaluation of u at time t^n. Next we apply the backward Euler method to the time derivative obtaining:

$$\frac{\partial u}{\partial t}(t^{n+1}) \sim \frac{u^{n+1}-u^n}{\Delta t}.$$

Applying this to (1) we are left with the problem

$$u^{n+1} - \Delta t\nabla(D\nabla u^{n+1}) = -\Delta t I^{n+1} + \Delta t u^n.$$

For the finite dimensional approximation in space we first set up the variational formulation of our problem (1). Let $H^1(\Omega)$ be the Sobolev space over $\Omega \subset \mathbb{R}^2$. Then the variational formulation of (1) is

$$\begin{aligned}&\text{Given } u^0, w^0 \in L^2(\Omega) \text{ and } I \in L^2(\Omega \times (0,T))\\&\text{find } u^{n+1} \in V_h \text{ such that for all } t \in (0,T):\\&\int_\Omega u^{n+1}\phi_j + \Delta t \int_\Omega D\nabla u^{n+1}\nabla\phi_j = \Delta t \int_\Omega u^n\phi_j - \Delta t \int_\Omega I^{n+1}\phi_j, \forall \phi_j \in V_h\end{aligned} \tag{4}$$

for each $n = 0, \ldots, N-1$. Let $\mathcal{T}_h$ be a triangulation of Ω, which implies $\Omega = \bigcup_{K \in \mathcal{T}_h} K$. We define h as the maximum diameter of the triangles. For reasons of simplicity we assume that every triangle K can be obtained by applying a suitable invertible affine map to a reference triangle $\hat{K}$, thus $K = T_K(\hat{K})$. The corresponding finite element spaces are defined as

$$X_h^1 = \{v_h \in C^0(\Omega) | v_{h|K} \circ T_K \in \mathbb{P}_1, \forall K \in \mathcal{T}_h\}$$

where $\mathbb{P}_1$ is the space of polynomials of degree less than or equal to one. For a more detailed introduction to finite element methods see (Quarteroni and Valli, 1997).
For the approximation of our problem we apply the Galerkin method selecting the finite dimensional space $V_h = X_h^1$ and its basis $\{\varphi_j(x) | j = 1, \ldots, N_h\}$, where N_h is the dimension of V_h. With this we can set

$$u_h^n = \sum_{i=1}^{N_h} u_{h,i}^n \varphi_i(x).$$

Following the Green formula of integration we can deduce from the variational formulation (4):

$$\begin{aligned}&\sum_{i=1}^{N_h} u_{h,i}^{n+1} \sum_{K \in \mathcal{T}_h} \int_K \varphi_i(x)\varphi_j(x)dx + \Delta t \sum_{i=1}^{N_h} u_{h,i}^{n+1} \sum_{K \in \mathcal{T}_h} \int_K (\nabla\varphi_i(x))^T D \nabla\varphi_j(x)dx =\\&\Delta t \sum_{i=1}^{N_h} u_{h,i}^{n} \sum_{K \in \mathcal{T}_h} \int_K \varphi_i(x)\varphi_j(x)dx - \Delta t \sum_{i=1}^{N_h} I_h^{n+1} \sum_{K \in \mathcal{T}_h} \int_K \varphi_i(x)\varphi_j(x)dx.\end{aligned} \tag{5}$$

Defining the mass and stiffness matrix as $M = (m_{ij})$ and $S = (s_{ij})$ with

$$m_{ij} = \sum_{K \in \mathcal{T}_h} \int_K \varphi_i(x)\varphi_j(x)dx \quad \text{and} \quad s_{ij} = \sum_{K \in \mathcal{T}_h} \int_K (\nabla\varphi_i(x))^T D \nabla\varphi_j(x)dx$$

the finite element approximation reads

$$(M + \Delta t S)u^{n+1} = Mu^n - \Delta t M I^{n+1}.$$

The integrals in mass and stiffness matrix are evaluated by a third order Gauss rule. The equation for the recovery variable w in (3) can be solved explicitly as follows

$$\begin{aligned} w(t) &= \left(w(t_0) + \int_{t_0}^{t} \eta_2 (u - u_0) \exp\left(\int_{t_0}^{x} \eta_2 \eta_3 \mathrm{d}\xi \right) \mathrm{d}x \right) \exp\left(- \int_{t_0}^{t} \eta_2 \eta_3 \mathrm{d}x \right) \\ &= \frac{u - u_0}{\eta_3} + \left(w(t_0) - \frac{u - u_0}{\eta_3} \right) \exp\left(-\eta_2 \eta_3 (t - t_0) \right). \end{aligned}$$

For the full discretisation of the model (1) - (3) we use an implicit-explicit (IMEX) scheme to advance from t^n to t^{n+1}: the recovery variable w^{n+1} is updated by solving explicitly (after linearization around u^n) equation (3) in $(0, \Delta t)$ and plugged into the expression of $I(u, w)$ for the computation of u^{n+1}. The overall procedure can be summarized as follows

Given u^n and w^n,

$$\begin{aligned} &\text{update:} \quad w^{n+1} = \frac{u^n - u_0}{\eta_3} + \left(w^n - \frac{u^n - u_0}{\eta_3} \right) \exp\left(-\eta_2 \eta_3 \Delta t \right) \\ &\text{update:} \quad I^{n+1} = I(u^n, w^{n+1}) \\ &\text{solve:} \quad A u^{n+1} = M u^n - \Delta t M I^{n+1} \end{aligned}$$

where $A := M + \Delta t S$, where M and S are the classical finite elements mass and stiffness matrices.

REFERENCES

Quarteroni, A. and Valli, A. (1997). *Numerical Approximation of Partial Differential Equations* (Springer-Verlag Berlin Heidelberg), 2 edn.

2 SUPPLEMENTARY TABLES AND FIGURES

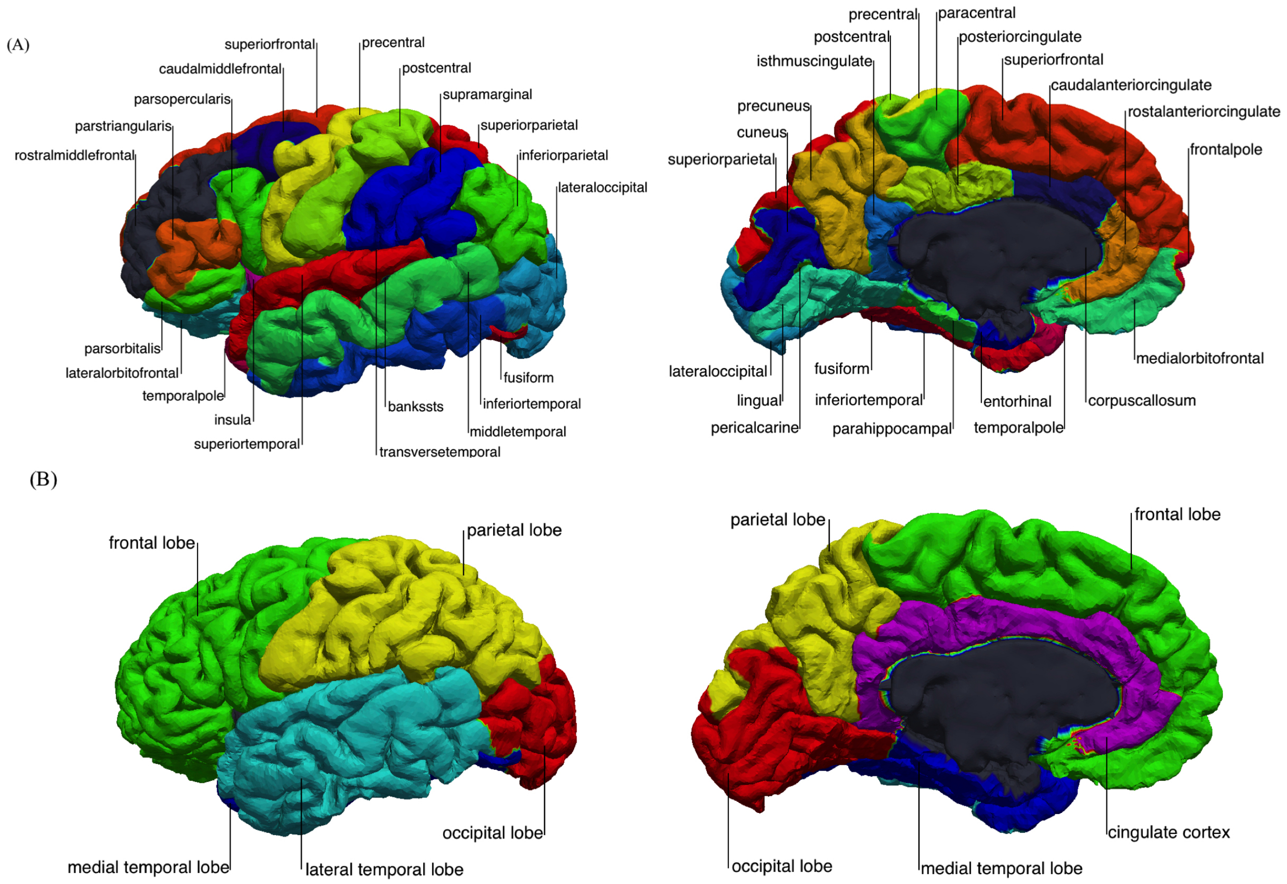

Supplementary Figure 1. The structural compartments (A) and the location of the lobes (B) on the lateral and medial surface of the cerebral cortex (left hemisphere).
